# CoFe_2_O_4_@rGO as a Separator Coating for Advanced Lithium–Sulfur Batteries

**DOI:** 10.1002/smsc.202300045

**Published:** 2023-06-27

**Authors:** Yan Li, Jiabing Liu, Xingbo Wang, Xiaomin Zhang, Ning Chen, Lanting Qian, Yongguang Zhang, Xin Wang, Zhongwei Chen

**Affiliations:** ^1^ South China Academy of Advanced Optoelectronics & International Academy of Optoelectronics at Zhaoqing South China Normal University Guangzhou 510006 China; ^2^ State Key Laboratory of Reliability and Intelligence of Electrical Equipment School of Materials Science and Engineering Hebei University of Technology Tianjin 300130 China; ^3^ Canadian Light Source Saskatoon S7N 2V3 Canada; ^4^ Department of Chemical Engineering University of Waterloo 200 University Ave. W Waterloo Ontario N2L 3G1 Canada; ^5^ School of Materials Science and Engineering State Key Laboratory of Reliability and Intelligence of Electrical Equipment Hebei University of Technology Tianjin 300130 China

**Keywords:** catalytic conversion, CoFe_2_O_4_@rGO, electrochemical performance, lithium–sulfur batteries, modified separators

## Abstract

Lithium–sulfur (Li–S) batteries are hindered by the undesired shuttle effect and sluggish electrochemical conversion kinetics. Herein, a well‐designed CoFe_2_O_4_@reduced graphene oxide (CFO@rGO) composite is used to modify the separator to develop a multifunctional polysulfide barrier. Density functional theory (DFT) calculations confirm that highly electronegative oxygen ions in CFO tend to bond with transition metal (TM) ions at octahedral (O_h_) sites, which induces the formation of Fe—S and Co—S bonds between CFO and polysulfides. This indicates that CFO can effectively anchor polysulfides. Furthermore, the low Li_2_S decomposition energy barrier and Li^+^ diffusion energy barrier reveal that CFO can accelerate the redox reaction kinetics of sulfur species. Electronic structure calculations speculate that the low‐energy barrier can be attributed to the electron‐hopping phenomenon between TM ions of different valence states at O_h_ sites. Benefiting from these advantages, a CFO@rGO/PP separator demonstrates satisfactory cycling performance (0.087% capacity decay rate at 2C with 500 cycles) and superb rate performance (686 mAh g^−1^ at 5C). This work provides a valuable reference for future research on spinel‐type materials as electrocatalysts for Li–S batteries.

## Graphical abstract

CoFe_2_O_4_ (CFO) nanoparticles are beneficial for the anchoring and catalytic conversion of polysulfides. Furthermore, reduced graphene oxide (rGO) can produce efficient ionic conductive channels and prevent the aggregation of CFO nanoparticles. Therefore, a CFO@rGO/PP separator can effectively suppress the shuttle effect and accelerate the reaction kinetics to significantly improve the cycling stability of lithium–sulfur batteries.

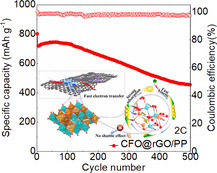

## 1 Introduction

In contemporary society, the booming development of electric vehicles, drones, popular consumer electronics, and smart grids has led to a strong demand for energy storage technologies.^[^ ^1^, ^2^, ^3^, ^4^ ^]^ Benefiting from the high theoretical capacity of sulfur (1675 mAh g^−1^), lithium–sulfur (Li–S) batteries are considered to satisfy emerging energy‐storage demands.^[^ ^5^ ^]^ In addition, sulfur has attractive commercialization characteristics, such as its low cost, environmentally friendliness, and natural abundance.^[^ ^6^, ^7^, ^8^, ^9^ ^]^ Nevertheless, the application of Li–S batteries faces a significant challenge of the shuttle effect caused by the high solubility and mobility of lithium polysulfides (LiPSs), which leads to fast capacity fading. In addition, the sluggish conversion kinetics of LiPSs leads to inferior rate performance.^[^ ^10^, ^11^, ^12^ ^]^

Numerous strategies have been proposed to target the above problems to alleviate the shuttle effect of LiPSs.^[^ ^13^ ^]^ Specifically, optimizing the cathode structure by designing suitable composite as the sulfur host material can effectively immobilize polysulfides.^[^ ^14^, ^15^ ^]^ However, such modifications on the cathode side will inevitably reduce the energy density of the battery and is accompanied by complicated and expensive fabrication processes.^[^ ^16^ ^]^ Given this, the modification of the separator is an effective and simple method to intercept the diffusion of polysulfides, which is expected to bring new vitality to Li–S batteries.^[^ ^17^, ^18^, ^19^, ^20^ ^]^ In early studies, many carbon materials (e.g., reduced graphene oxide, acetylene black, and multiwalled carbon nanotubes) were widely used for separator modification because of their own advantages of light weight, high electrical conductivity, and ease of processing.^[^ ^21^ ^]^ However, the interaction between nonpolar carbon and polar polysulfides is not sufficient to effectively immobilize polysulfides in the cathode side.^[^ ^22^ ^]^ To strengthen the adsorption capacity of polysulfides, polar materials such as metal oxides (CeO_2_^[^ ^23^ ^]^and V_2_O_5_^[^ ^24^ ^]^) and metal sulfides (ZnS^[^ ^25^ ^]^ and MoS_2_^[^ ^26^ ^]^) have been used to modify the separators.^[^ ^27^ ^]^ Beyond that, some multielement metal oxides containing two metal ions and oxygen anions, such as CoMoO_4_, MnFe_2_O_4_, and LiV_3_O_8_, have stronger interactions with polysulfides.^[^ ^28^, ^29^, ^30^, ^31^ ^]^ Among them, spinel‐type binary transition metal oxides (AB_2_O_4_) with excellent properties such as natural abundance, ecofriendliness, and chemical stability have been proven to be widely used in fields such as supercapacitors and fuel cells.^[^ ^32^, ^33^ ^]^ However, few studies have reported the internal structure of spinel, which facilitates a better understanding of its catalytic mechanism in Li–S batteries.^[^ ^34^ ^]^ In terms of its structure, metals located at octahedral (O_h_) sites are preferentially exposed to the spinel oxide surface, while metals located at tetrahedral (T_d_) sites are almost undetectable near the surface.^[^ ^35^ ^]^ Therefore, the occupancy of metals at O_h_ sites has a certain degree of influence on its electrical conductivity and catalytic properties.

CoFe_2_O_4_ (CFO) belongs to a cubic inverse spinel structure characterized by Co^2+^ occupying half of the O_h_ sites, Fe^3+^ occupying the other half of the O_h_ sites, and all T_d_ sites.^[^ ^36^ ^]^ In the structure of CFO, electron hopping between two metals of different valence states at the O_h_ sites gives it satisfactory catalytic properties and good electrical conductivity.^[^ ^37^ ^]^ Meanwhile, the ionic bonds formed by metal–oxygen in the CFO structure are strongly polarized, which facilitates the adsorption of the polar intermediate LiPSs. Therefore, the rational design of CFO has much significance for advanced Li–S batteries.

Herein, we fabricated a CFO nanoparticle@reduced graphene oxide (CFO@rGO) composite by a simple hydrothermal reaction and annealing process as a separator modifier for Li–S batteries. The introduced rGO enhances the electrical conductivity and prevents the agglomeration of CFO nanoparticles for a facile ion/electron transfer and fully exposed active interface. More importantly, a series of computational and experimental characterizations demonstrated that the diverse metal sites and highly polar negative ions of CFO can achieve anchoring of dissociated polysulfides and accelerating cell kinetics. Attributed to these advantages, Li–S cell with the CFO@rGO/PP separator exhibits a high initial capacity of 1160 mAh g^−1^ at 0.2C and a satisfactory capacity decay rate (only 0.087% per cycle for 500 cycles at 2C).

## 2 Results and Discussion

**Figure 1:**
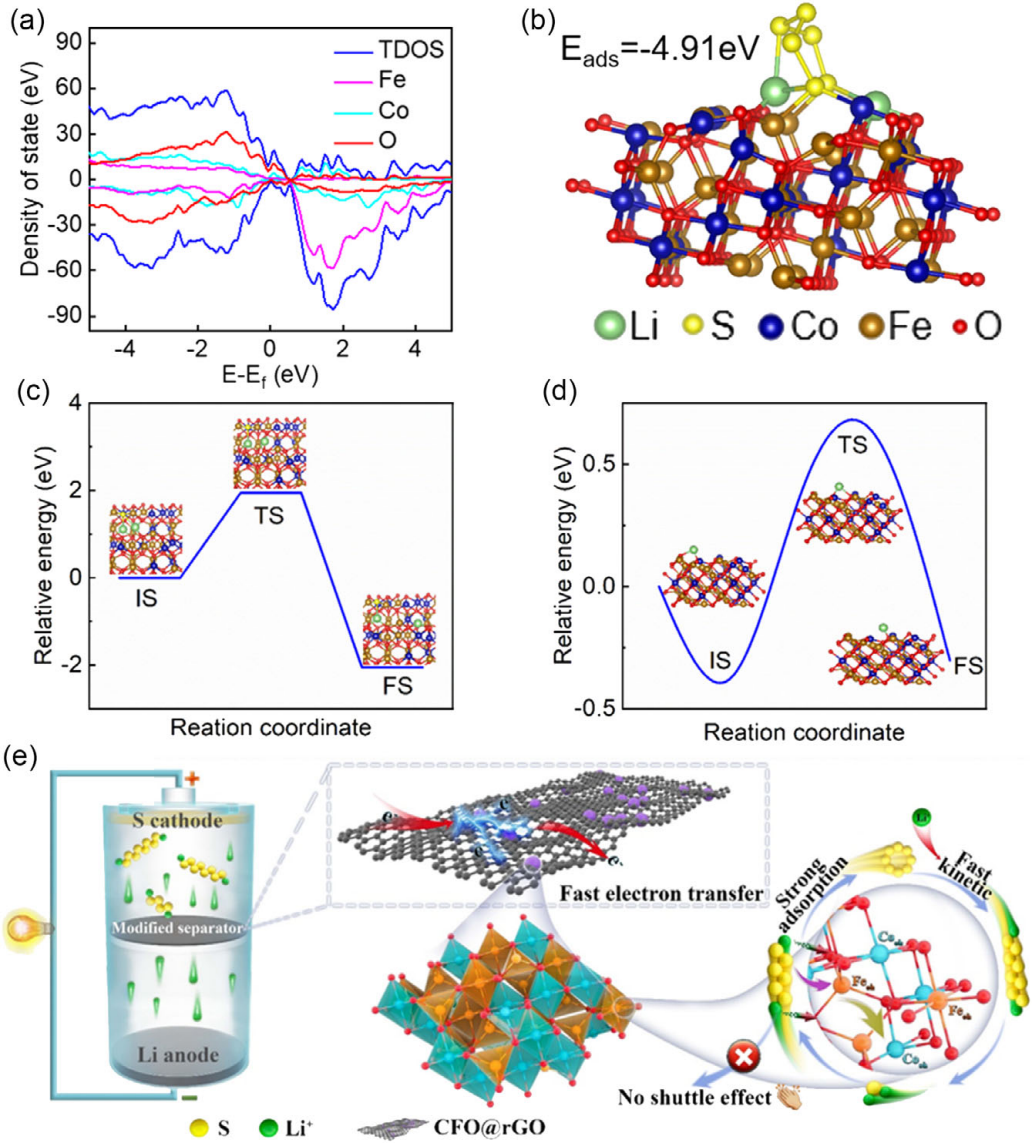
a) DOS profiles of CFO. b) Chemical interactions between Li_2_S_6_ and CFO. c) The transition state of Li_2_S decomposition. d) Diffusion profiles of Li^+^ on surface of CFO (311). e) Schematic diagram of Li–S batteries designed with CFO@rGO/PP separators.

Density functional theory (DFT) calculations were initially used to evaluate the adsorption and catalytic conversion abilities to LiPSs of CFO. The density of states (DOS) in **Figure** **1**a shows the half‐metallic properties of CFO, which facilitates fast electron transfer. Figure 1b shows the stable geometric configuration of Li_2_S_6_ adsorption on the CFO (311) face. The results indicate large binding energy of −4.91 eV for Li_2_S_6_ binding to CFO, which can indicate that CFO contributes to strong chemical sulfur fixation and high‐effective inhibition of the shuttle effect. The decomposition and conversion of Li_2_S during charging is a key process that affects the reaction kinetics of Li–S batteries. To investigate the main reason why CFO promotes the decomposition of Li_2_S, we developed a molecular model of Li_2_S and CFO, and the calculated decomposition energy barrier of Li_2_S on the surface of CFO (1.95 eV) is shown in Figure 1c. The low‐energy barrier ensures that CFO can effectively catalyze the breaking of the Li–S bond and thus faster Li_2_S decomposition kinetics. Remarkably, the good catalysis of polysulfides by CFO is due to the complex cation occupation at the O_h_ site. Additionally, Figure 1d depicts the Li^+^ diffusion pathways and the corresponding geometric configurations of CFO on the (311) surface. The lower diffusion potential barrier energy (1.07 eV) confirms the facilitation of Li^+^ transfer. Motivated by the DFT predictions, we prepared CFO@rGO composite by hydrothermal reaction and annealing process for use as a separator modifier to facilitate the realization of high‐performing Li–S batteries (Figure 1e).

**Figure 2:**
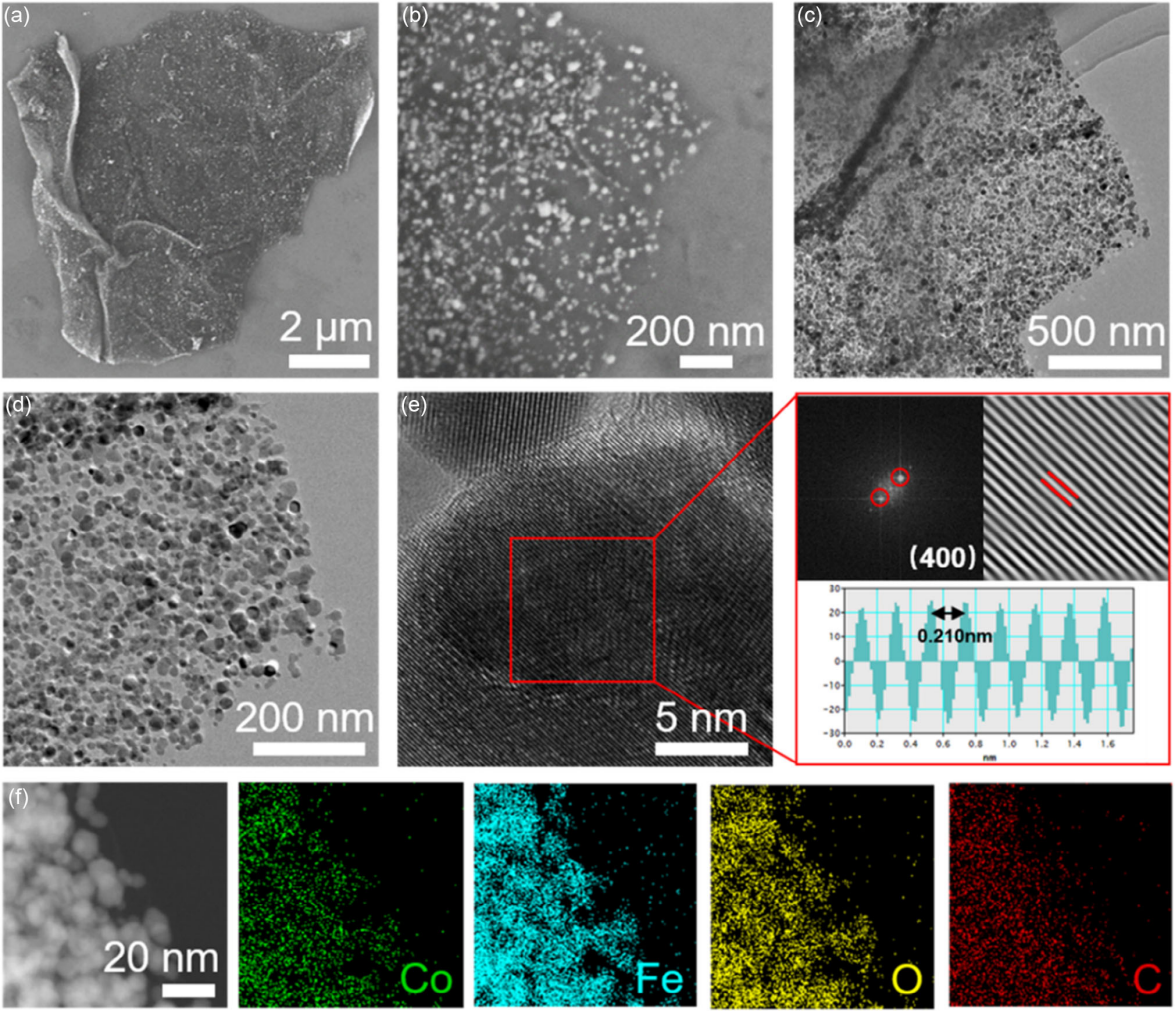
a,b) SEM and c,d) TEM images of CFO@rGO. e) High‐angle annular dark‐field scanning transmission (HAADF‐STEM) element mapping images of CFO@rGO. f) HRTEM images of the CFO@rGO.

Scanning electron microscopy (SEM) and transmission electron microscopy (TEM) have been studied to understand the morphological characteristics of the prepared materials. As shown in Figure S1, Supporting Information, the hollow CFO nanospheres have a rough surface and a diameter of about 150 nanometers. As shown in the CFO@rGO composites illustrated in **Figure** **2**a–d, CFO was grown in situ as nanoparticles on rGO with diameters ranging from 10 to 30 nm. This allows the CFO to expose more active sites. The large surface area and functional groups of the rGO not only provide sufficient nucleation sites for CFO growth, but also anchor the CFO particles and limit their further growth. Furthermore, the attached CFO nanoparticles act as spacers preventing the direct restacking of rGO. Consequently, a high active surface area of reduced graphene oxide could be maintained. The high‐resolution transmission electron microscope (HRTEM) image in Figure 2e clearly observes the lattice stripes belonging to CFO nanoparticles, corresponding to the (400) crystal plane. Meanwhile, the presence of a carbon matrix is also confirmed by the surrounding amorphous region. The clearly visible diffraction rings in the selected‐area electron diffraction (SAED) image of Figure S2, Supporting Information, illustrate the polycrystalline character of the CFO@rGO composite. Furthermore, Figure 2f shows the distribution of CFO nanoparticles on the rGO substrate, which is relatively homogeneous.

**Figure 3:**
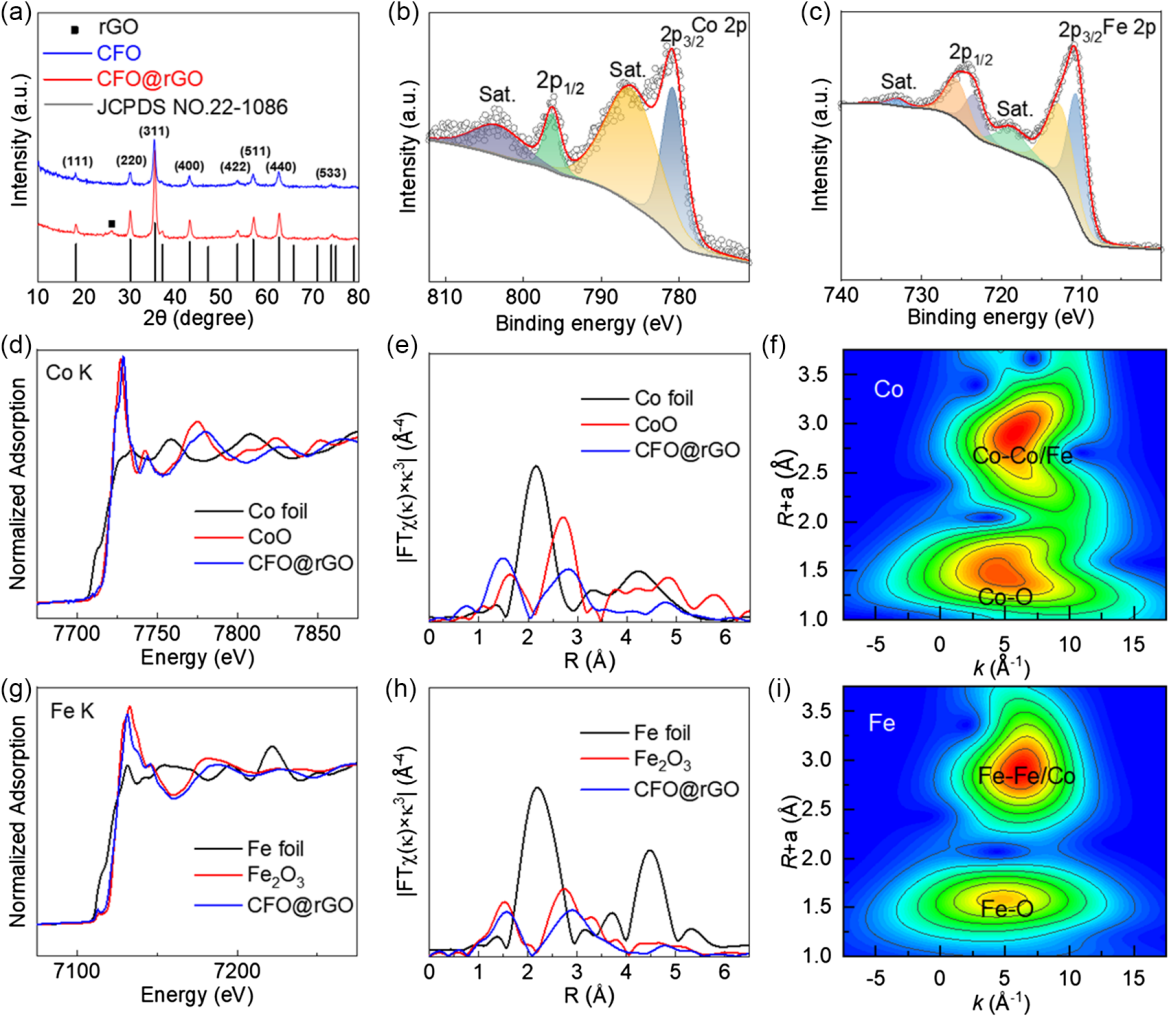
a) XRD patterns of CFO and CFO@rGO. b) Fe 2p and c) Co 2p spectra of CFO@rGO. d,g) X‐ray absorption near edge structure (XANES) spectra of Co (d) and Fe (g) K‐edge of CFO@rGO. e,h) EXAFS spectra in R space of Co (e) and Fe (h) K‐edge of CFO@rGO. f,i) Wavelet transforms for the EXAFS signals of Co (f) and Fe (i) k‐edge of CFO@rGO.

The X‐ray diffraction (XRD) patterns of CFO and CFO@rGO are summarized in **Figure** **3**a. The XRD patterns of CFO and CFO@rGO are matched to the CoFe_2_O_4_ phase (JCPDS No. 22‐1086),^[^ ^38^ ^]^ In addition, CFO@rGO shows a broad reflection (indicated by black squares) belonging to rGO at ≈26°. Since no other distinct diffraction peaks are found in the XRD pattern, it can be determined that CFO@rGO consists of CFO and rGO phases. Raman spectra of CFO@rGO are shown in Figure S3, Supporting Information, where 1353 cm^−1^ (D band) and 1581 cm^−1^ (G band) are two well‐known carbon Raman bands associated with the disordered carbon and graphitized carbon, respectively. The higher *I*_D_/*I*_G_ (1.04) ratio of CFO@rGO can confirm the presence of defective graphitic structures and highly porous structure in the rGO, which facilitates fast electron transfer during electrochemical reaction. In order to deeply explore in the surface chemical state of CFO@rGO, X‐ray photoelectron spectroscopy (XPS) was conducted. As detected by XPS results, the four obvious peaks correspond to Fe 2p (711.14 eV), Co 2p (781.02 eV), C 1s (284.94 eV), and O 1s (530.12 eV), respectively (Figure S4a, Supporting Information).^[^ ^39^ ^]^ As illustrated in Figure 3b, the Co 2p spectrum of the CFO@rGO composite consists of Co 2p_3/2_ (787.20 eV), Co 2p_1/2_ (803.48 eV), and two satellite peaks are located at 787.20 and 803.48 eV, respectively, indicating the presence of Co^2+^ in CFO@rGO. As shown in Figure 3c, two peaks appearing at 710.68 and 712.78 eV can be attributed to Fe 2p_3/2_, and the peaks at 723.38 and 725.88 eV can be attributed to Fe 2p_1/2_, accompanied by two satellite peaks at 718.7 and 733.4 eV, respectively, which prove the presence of Fe^3+^. It is worth mentioning that the peaks at 710.68 and 723.38 eV appear due to the binding of Fe^3+^ to the O_h_ of oxygen, while 712.78 and 725.88 eV are due to the binding of Fe^3+^ to the T_d_ of oxygen.^[^ ^40^ ^]^ The C 1s spectrum of CFO@rGO in Figure S4b, Supporting Information, is divided into three separate peaks at 284.77, 286.11, and 289.13 eV, regarded as C═C, C—O, and O—C═O bonds, respectively. Figure S4c, Supporting Information, can be well observed for the deconvolution of the O 1s signal. The peak at 530.3 eV corresponds to the O^2−^ ions in the transition metal oxide. Simultaneously, the peaks at 531.9 and 533.5 eV represent hydroxyl oxygen and surface‐adsorbed carbonyl oxygen, respectively.

To gain more insight into the valence and coordination environments of Co and Fe atoms in CFO@rGO, we performed X‐ray absorption fine structure (XAFS) measurements. As shown in Figure 3d,e, the near‐edge absorption of CFO@rGO is similar to that of the metal oxide foil used as a comparison, so it can be assumed that the valence states of Co and Fe are +2 and +3, respectively. Furthermore, the coordination environments of Co and Fe were investigated by Fourier‐transform extended X‐ray absorption fine structure (EXAFS) as an indication of Co and Fe occupation at T_d_ and O_h_ sites. In Figure 3f, the peaks of CFO@rGO at ≈1.5 and ≈3 Å can be identified as being due to the Co—O bond and Co—Co/Fe bond, respectively. In Figure 1g, the peaks of CFO@rGO at ≈1.5 Å, respectively, can be identified as Fe—O bonds. The peaks at ≈3 and ≈3.5 Å are caused by Fe—Co and Fe—Fe bonds, respectively.^[^ ^41^, ^42^ ^]^ This can also be observed more visually in the wavelet transform (WT) of the *k* ^3^‐weighted EXAFS in Figure 3h,i. The least‐squares refinement results show a detailed coordination number profile, further demonstrating that Co is present only in the O_h_ environment, while Fe is present in the T_d_ and O_h_ environments (Figure S5, S6 and Table S1, S2, Supporting Information). The porosity, specific surface area, and pore size of CFO@rGO were investigated thoroughly by N_2_ adsorption–desorption test (Figure S7, Supporting Information). The typical I/IV type isotherm indicates that the CFO@rGO sample has a mesoporous structure. The pore size distribution curves of Barret–Joyner–Halenda (BJH) are given in Figure S7b, Supporting Information. The large surface area (116.69 m^2^ g^−1^) and pore volume (0.371 cm^3^ g^−1^) possessed by CFO@rGO will provide many active sites that can strongly adsorb polysulfides, resulting in excellent performance of the cell in electrochemical reaction.

**Figure 4:**
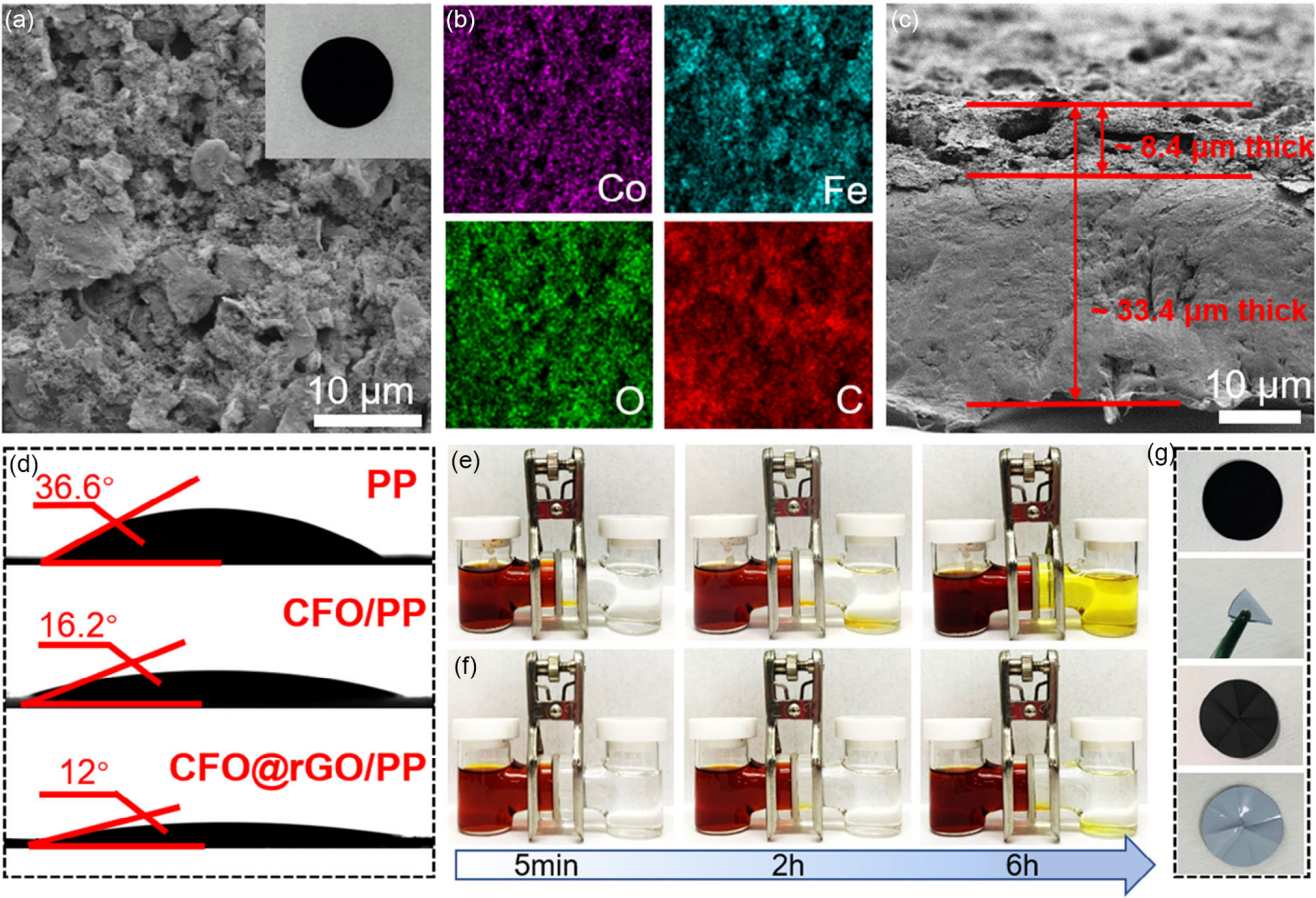
a) SEM image and photograph (inset) of CFO@rGO/PP separator. b)EDS elemental mapping. c) Cross‐sectional SEM image. d) Photograph of the contact angle of the electrolyte drop on different separator surfaces. e,f) Polysulfide permeation tests using H‐shape cells with the PP (e) and CFO@rGO/PP (f) separator. g) The kneading/spreading test of CFO@rGO/PP separator.

As can be visualized from **Figure** **4**a and S8, Supporting Information, the pristine PP separator possesses a large number of nanoscale pores, while the CFO@rGO/PP separator has a porous structure. In comparison, the porous structure can provide a better path for electrolyte penetration and lithium ion transport. The mapping images in Figure 4b of CFO@rGO/PP separator exhibit the uniform distribution of elements Co, Fe, O, and C on the separator surface. Thus, it can be assumed that the CFO@rGO/PP separator not only immobilizes the polysulfide, while acting as an upper‐current collector to convert the polysulfide. As shown in Figure 4c, the interface of coating layer and separator was tight, and the overall thickness of the CFO@rGO/PP separator (including the 25 μm PP layer) is ≈33.4 μm. It is well known that the wettability of the separator and the liquid electrolyte is associated with the resistance of the cell. As shown in Figure 4d, the contact angles of the PP and CFO/PP separators are 36.6° and 16.2°, respectively, while the contact angle of the CFO@rGO/PP separator is 12°. The difference in contact angle is a great proof that the CFO@rGO coating is effective in improving the wettability of the electrolyte, which can speed up the transport of lithium ions and reduce the interfacial resistance. The successful interception of polysulfides by the CFO@rGO/PP separator was verified based on visual experiments of obstructing Li_2_S_6_ (Figure 4e,f). Figure 4g shows that the modified separator does not flake even after multiple folding and still maintains its structural integrity, which further proves that a very tight relationship was established between the CFO@rGO and PP separator surface.

**Figure 5:**
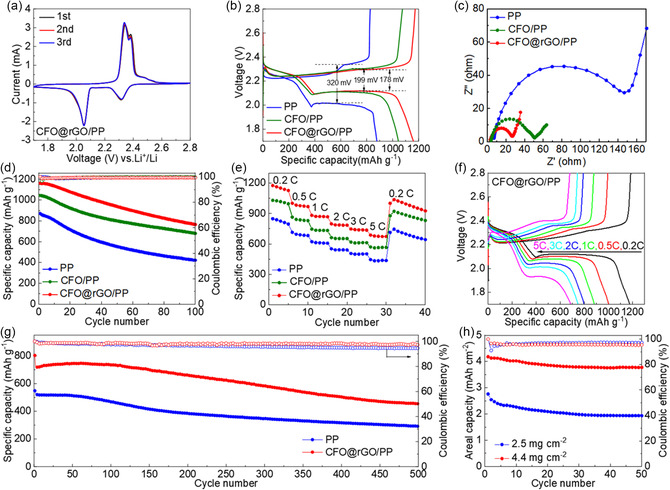
a) CV profiles at 0.1 mV s^−1^ of CFO@rGO/PP separator. b) Charge/discharge voltage curves at 0.2C, c) EIS before cycling, d) cycling performances at 0.2C, e) rate capabilities, f) multirate voltage profiles, and g) cycling performances at 2C of Li–S cells with different separators. h) Cycling performances at high sulfur loadings.

**Figure** **5**a shows the cyclic voltammetry (CV) profiles of the cell using the CFO@rGO/PP separator in the first three cycles. There are two cathodic peaks at 2.31 and 2.05 V, representing the reduced conversion of sulfur to long‐chain LiPSs and followed by the reconversion to Li_2_S_2_/Li_2_S, respectively.^[^ ^43^, ^44^ ^]^ Subsequently, upon charging, two anodic peaks around at 2.34 and 2.38 V are related to the reverse oxidation of polysulfides to the neutral S_8_.^[^ ^45^ ^]^ It can be observed that the peaks are almost identical in shape and intensity after several scans, indicating good cycling stability and excellent reversibility. In addition, compared to Figure S9, Supporting Information, it can be seen that CFO@rGO/PP has the highest peak current and the largest coverage area. The above results indicate that the presence of CFO@rGO coating enhances the redox kinetics of the cell. Figure 5b illustrates the galvanostatic discharge/charge curves of the cells utilizing different separators for the first cycle at 0.2C. It can be seen that the cell with CFO@rGO/PP separator has the smallest polarization. The kinetics of Li–S batteries can be reflected by electrochemical impedance spectroscopy (EIS) analysis. Nyquist plots of the different samples before cycling are shown in Figure 5c. It is clear that the *R*_ct_ value of the CFO@rGO separator sample is much lower than the other samples, which favors faster redox kinetics and reduces the cell polarization. This can be corresponded to the voltage profile in Figure 5b.

Figure 5d shows cycling tests of the Li–S cells with different separators at 0.2C. The initial discharge capacities of the cells with PP, CFO/PP. and CFO@rGO/PP separators are 870, 1044, and 1160 mAh g^−1^, respectively. After that, the cell with CFO@rGO/PP separator still retained a capacity of 765 mAh g^−1^ at the 100th cycle, corresponding to a capacity retention rate of 65.9%. In contrast, the capacity of the cells with PP and CFO separators was maintained at 422 and 680 mAh g^−1^, respectively. It can be concluded that the presence of CFO@rGO coating improves the specific capacity and capacity retention of the cell, which also proves that the CFO@rGO composite not only effectively inhibits the diffusion of polysulfides but also contributes to the improvement of the utilization of the active material. Figure 5e shows that the cell with the CFO@rGO/PP separator possesses significantly higher specific capacities at 0.2, 0.5, 1, 2, 3, and 5C. The reason for the improved rate performance of the cell with CFO@rGO coating is due to the physical blocking and chemisorption of polysulfides by rGO and CFO, respectively, and the secondary current collection function possessed by rGO. Apart from that, the reversible capacity of the cell with the CFO@rGO/PP separator reached about 1002 mAh g^−1^ after abruptly switching the current rate of 0.2C. As illustrated in Figure 5f and S10, Supporting Information, the charge/discharge curves of CFO@rGO/PP consistently show a more stable and flatter charge/discharge plateau and minimal polarization. The above comparison results once again provide strong evidence that using CFO@rGO/PP as a separator for batteries can lead to good stability and reversibility. Figure 5d presents 500 continuous cycles of batteries with PP and CFO@rGO/PP separators at 2C. The CFO@rGO/PP separator exhibits an impressive capacity (455.6 mAh g^−1^) after 500 cycles, which equates to a low fading rate of 0.087% per cycle. It shows a higher discharge capacity and lower capacity attenuation than cells with PP separators. With excellent cycling stability at 2C, it can be assumed that this battery is well suited for use at high current densities.

**Figure 6:**
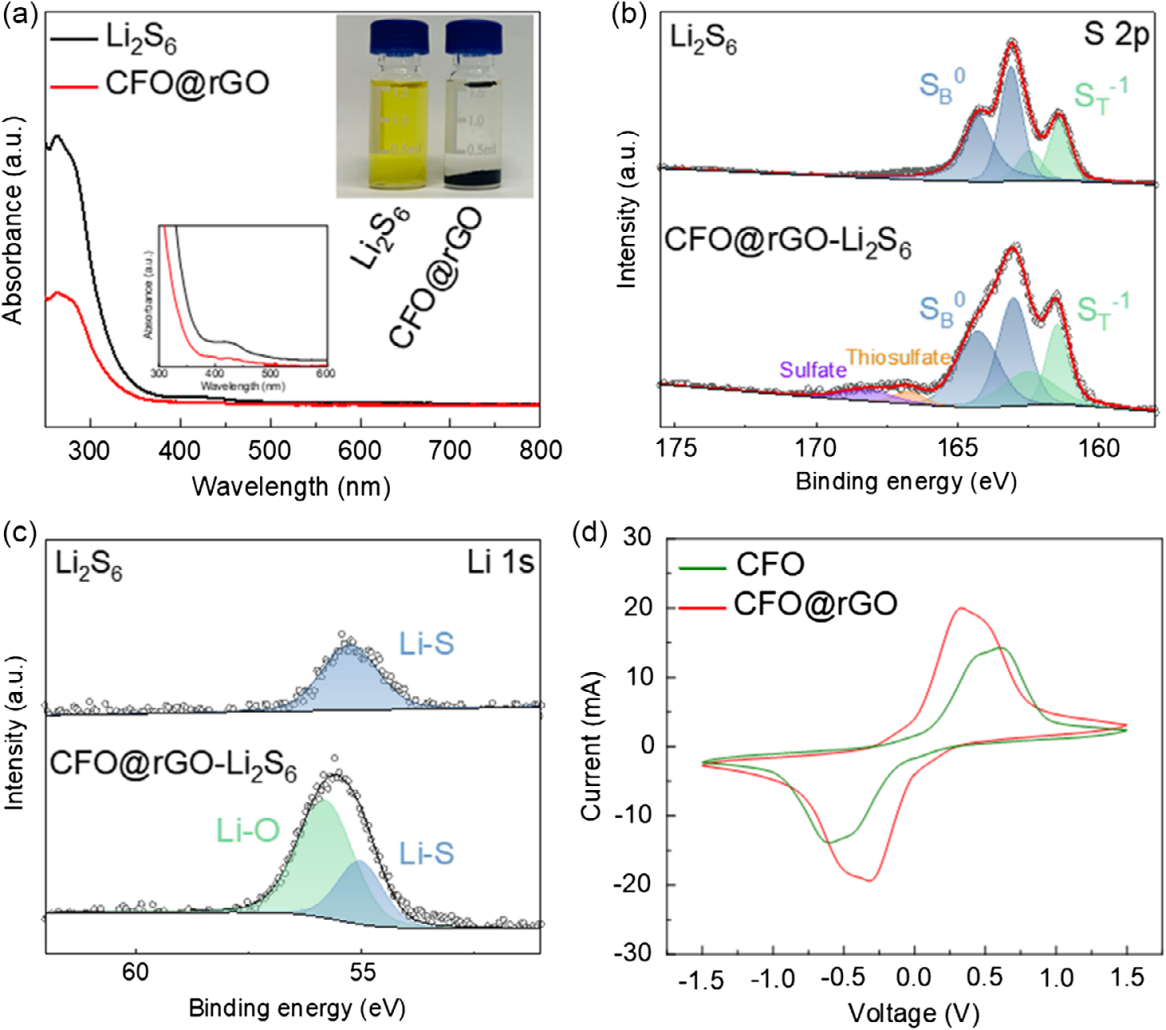
a) UV–vis absorption spectrum and adsorption properties (inset) of the CFO@rGO in Li_2_S_6_ solution. b) S 2p and c) Li 1s XPS spectra of Li_2_S_6_ and CFO@rGO‐adsorbed Li_2_S_6_, respectively. d) CV curves of Li_2_S_6_ symmetric cells.

The ultraviolet/visible absorption measurement is carried out to investigate the Li_2_S_6_ adsorption effect of CFO@rGO. An orange solution of Li_2_S_6_ contains CFO@rGO powder, which turns nearly clear after 12 h of standing (inset of **Figure** **6**a). The intensity of the characteristic peaks of CFO@rGO at 263.4 and 426.6 nm is much weaker than that of Li_2_S_6_, which shows the significant absorption of LiPSs by CFO@rGO. Consequently, we are confident that CFO@rGO/PP separator would be an ideal ion sieve that both allows the free passage of lithium ions and restricts the shuttling of polysulfides. XPS analysis is used to reveal the reaction mechanism between CFO@rGO and polysulfides. For the spectrum of S 2p in Figure 6b, peaks at 161.4 and 162.4 eV correspond to terminal sulfur (S_T_^−1^) and peaks at 163 and 164.3 eV related to bridging sulfur (S_B_^0^).^[^ ^46^ ^]^ Furthermore, the signals at 166.7 and 168.2 eV correspond to the formation of sulfate species formed by the strong chemical reaction between CFO@rGO and the polysulfides. Figure 6c shows that the Li 1s of Li_2_S_6_ shows a peak at 55.2 eV, signifying Li—S bonding. After adsorption, a new peak appears at 55.8 eV, signifying Li—O bond, which implies a chemical interaction between CFO@rGO and Li_2_S_6_. To accurately reflect the catalytic effect of the material on polysulfide conversion, symmetric Li_2_S_6_–Li_2_S_6_ cells were fabricated with CFO and CFO@rGO as electrodes, respectively. As shown in Figure 6h, both electrodes exhibited a current response, which was the result of Li_2_S_6_ oxidation and reduction. Among them, the CFO@rGO electrode has the highest high current response and the lightest polarization, which indicate that CFO@rGO can more effectively catalyze the polysulfide conversion and improve the reaction kinetics of the cell.

**Figure 7:**
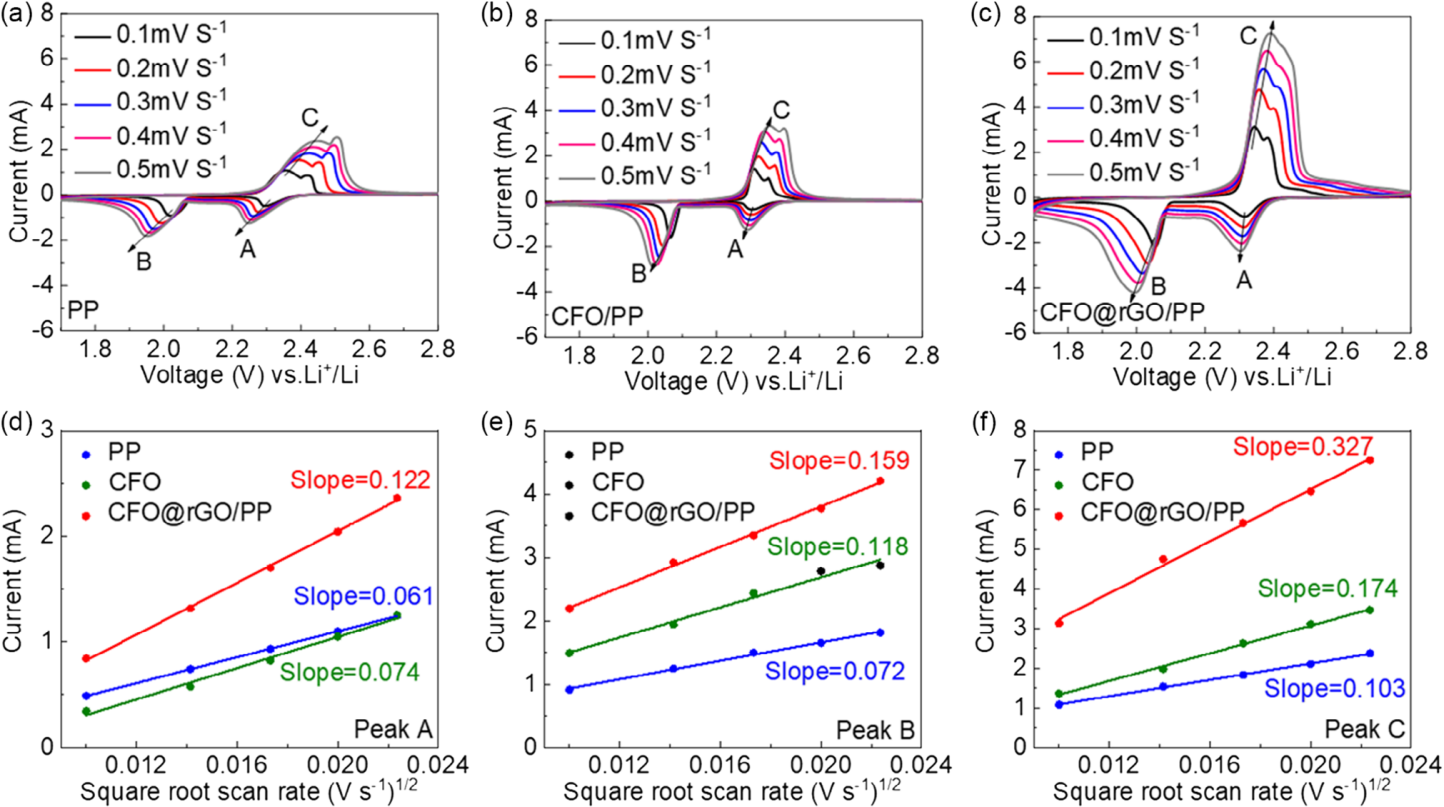
a–c) Representative voltammograms of Li–S cells with PP (a), CFO/PP (b), and CFO@rGO/PP (c) separators and d–f) corresponding linear fit plots.

The Li^+^ ion diffusion coefficient ($D_{{\text{Li}}^{+}}$) of the Li–S system is also a means to verify the catalytic performance of the CFO@rGO composite. As shown in **Figure** **7**a–c, CV tests are performed for cells assembled with PP, CFO/PP, and CFO@rGO/PP separators at different scan speeds. It is observed that the oxidation and reduction peaks show similar shifts with increasing scan rate, which is due to the polarization associated with ion transfer. Figure 7d–f is obtained according to the Randles–Sevick equation, where the slope of *I*_p_ with respect to *ν* ^1/2^ is proportional to the magnitude of $D_{{\text{Li}}^{+}}$.^[^ ^47^ ^]^ The highest slope value of CFO@rGO/PP implies that the presence of CFO@rGO coating gives the cell superior diffusion kinetics throughout the charging and discharging process.

**Figure 8:**
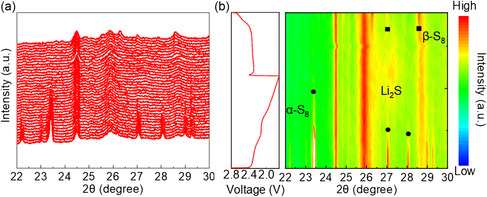
a) In situ XRD patterns of Li–S cell with CFO@rGO/PP separator in the initial cycle, and b) the corresponding discharge/charge curves (left) and contour plots (right).

To further investigate the details of the phase transition occurring during the electrochemical reaction, an in situ XRD test is performed. From **Figure** **8**a, we can see that at the beginning of the lithiation process, the more obvious crystalline α‐S_8_ characteristic peaks existing around 23° and 29° gradually disappear, which indicates that the reaction between S_8_ and lithium ions has been fully carried out. Subsequently, during continuous discharge, a broad peak centered at 2*θ* ≈ 27.2° appeared that can be assigned to Li_2_S (JCPDF No. 077‐2145). It can be seen very clearly in the contour plot of Figure 8b that the peak intensity of this peak increases until the end of the discharge. On the contrary, during the charging process, the peak representing Li_2_S can be observed to gradually weaken and disappears, which indicates the complete conversion of Li_2_S into Li polycrystals. At the end of the charging process, near 27° and 28.5°, not only the S signal is regenerated but also its intensity increases until the end of charging.^[^ ^48^, ^49^ ^]^ The process of desulfurization of lithium sulfide to form sulfur is a strong proof that the cell has good electrochemical reversibility. Thus, in situ XRD test reveals the interphase evolution of sulfur and demonstrates that the cell with CFO@rGO‐modified separator has a facilitating effect on the conversion of α‐S_8_ crystals to Li_2_S and the eventual access to the β‐S phase during the charging and discharging process.

**Figure 9:**
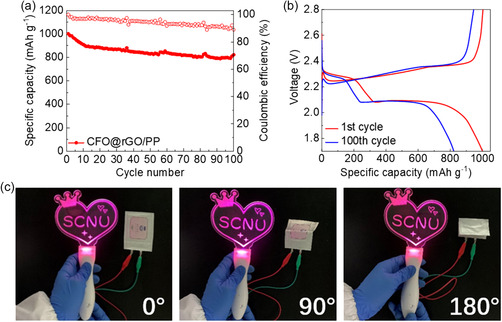
a) Cycling performance at 0.1C and b) charge/discharge curves of Li–S pouch battery with CFO@rGO/PP separator. c) Optical images of Li–S pouch battery with lighted sign at 0°, 90°, and 180° bends, respectively.

We assemble the Li–S soft‐package cell using CFO@rGO/PP separator to demonstrate its scalability potential. The first cycling capacity of the Li–S pouch cell with CFO@rGO/PP separator is 1001 mAh g^−1^ at 0.1C. Notably, the capacity retention rate remains at 81% even after 100 cycles (**Figure** **9**a). Furthermore, Figure 9b shows a relatively flat charge–discharge curve. The specific capacity of the pouch cell is slightly lower than that of the coin cell because of the difference in the thickness of the lithium metal foil and the cell structure. Encouragingly, even when the battery is bent at 180°, it does not affect the luminescence of the light sign with the “SCNU” pattern (Figure 9c).

To further compare the suppressing shuttle effect ability of the pristine and the CFO@rGO coating separator, the cells after 100 cycles at 1C are disassembled and analyzed using SEM. As illustrated in Figure S11, Supporting Information, the lithium anode surface of the CFO@rGO/PP separator sample exhibits relatively smooth, while the lithium anode surface of the PP separator sample exhibits severe corrosion. These results imply that the migration of polysulfides is well controlled by the CFO@rGO coating, which fully demonstrates the superiority of the CFO@rGO coating in absorbing dissolved polysulfides.

## 3 Conclusion

The CFO@rGO composite is developed through a simple hydrothermal reaction and annealing process to construct a unique multifunctional LiPSs barrier for advanced Li–S batteries. The highly conductive rGO can prevent CFO nanoparticles from agglomerating to provide sufficient active sites to capture polysulfides. Theoretical calculations and experimental analysis demonstrate that the CFO is an adsorbent for LiPSs, which can strongly anchor polysulfides via the formation of Co—S and Fe—S chemical bonds. Meanwhile, it is also an effective catalyst for promoting the sulfur redox kinetics. Attributed to these superiorities, the assembled cell using CFO@rGO/PP separator enables an exceptional cycling stability over 500 cycles with a negligible capacity decay of 0.087% per cycle at 2C and a superb rate capability (686 mAh g^−1^ at 5C). This work verifies the outstanding application potential of spinel oxides for Li–S battery systems, which can play a beneficial role in the future development of high‐efficiency energy storage.

## 4 Experimental Section

4.1

4.1.1

##### Preparation of CFO@rGO

Typically, FeCl_3_·6 H_2_O (1.35 g), CH_3_COONa·3 H_2_O (3.6 g), Co (CH_3_COO)_2_·4 H_2_O (0.27 g), and graphene oxide aqueous solution (28  mL, 10 mg mL^−1^) in 70 mL ethylene glycol were dissolved. At room temperature, the solution was stirred and sonicated until mixed homogeneously and then transferred to an autoclave and placed at 200 °C for 10 h. The resulting black precipitates were washed with ethanol and dried, then calcined in Ar flow at 2 °C min^−1^ to 500 °C, and held on for 2 h to further improve the conductivity of the carbon base. A black CFO@rGO was finally obtained by cooling to room temperature under argon flow. As a comparison, CFO was prepared by a one‐step hydrothermal method but without the addition of graphene oxide aqueous solution.

##### Separator Fabrication

Typically, the mixture of CFO@rGO/Super P/polyvinylidene fluoride (PVDF) (6:3:1 by weight) was ground in a mortar for 30 min, and then an appropriate amount of *N*‐methyl‐pyrrolidone (NMP) was added and ground. The well‐mixed slurry was coated on the commercial PP separator (Celgard 2400, thickness = 25 μm) by a doctor blade and dried in a vacuum oven at 60 °C to obtain the CFO@rGO/PP separator. As a comparison, CFO/PP separator was prepared by the same method, denoted as CFO/PP.

##### Preparation of Sulfur Cathode

The mixture containing sulfur (75 wt%) and carbon nanotube was ground for 30 min and then dropped into an appropriate amount of CS_2_ solution and continued to be ground until CS_2_ evaporated. Subsequently, the well‐mixed mixture was heated at 155 °C for 12 h in an Ar‐filled autoclave. The obtained product was denoted as sulfur/carbon nanotube (S/CNT) composite. The sulfur content of the S/CNT was 72.2% (Figure S12, Supporting Information). The electrode materials were ground with 70 wt% S/CNT composite, 20 wt% super P carbon, and 10 wt% PVDF in NMP. The fully ground slurry was pasted onto the carbon‐coated aluminum substrate and dried in air at 60 °C; finally, the Al foils were cut and weighed.

##### Adsorption Test

The Li_2_S and elemental S (5:1 by molar ratio) were dissolved in tetrahydrofuran (THF) and stirring at 60 °C for 48 h to obtain Li_2_S_6_ solution. 10 mg of CFO@rGO in 1.5 mL of as‐prepared Li_2_S_6_ electrolyte solution was soaked. The digital picture of virtually macroscopic adsorption of Li_2_S_6_ by adsorbent was taken with 12 h after vigorously shaking for 1 min.

##### Electrochemistry Measurement

Coin‐type (CR2032) cells were assembled in Ar atmosphere using S/CNT as the cathode and 0.6 mm‐thick lithium foil as the anode. Meanwhile, PP, CFO/PP, and CFO@rGO/PP were used as separators, respectively. The liquid electrolyte consisted of 1 mol L^−1^ lithium bis‐(trifluoromethyl sulfonyl) imide (LiTFSI) with 1 wt% lithium nitrate dissolved in a mixture of DOL and DME (1:1, v/v). Galvanostatic discharge–charge tests were conducted at a voltage window of 1.7–2.8 V using a Neware Battery Measurement System. The Bio‐logic VMP3 electrochemical workstation was used for CV and electrochemical impedance spectrometry (EIS) measurement (potential window: 1.7–2.8 V, frequency: 100 kHz to 10 mHz). All the electrochemical tests were conducted at 25 °C.

##### Characterization

SEM images were obtained with a Zeiss Ultra 55 scanning electron microscope. The morphology and structure of the samples were recorded on HRTEM (JEOL JEM‐2100). The crystal structures were characterized by XRD (BRUKER D8 ADVANCE). Raman spectra (Renishaw Invia) were acquired using an incident laser at 532 nm. Thermo Scientific K‐Alpha spectrometer was used for X‐ray photon spectroscopy (XPS) analysis with an X‐ray spot of 400 μm. Brunauer–Emmett–Teller (BET) method was utilized to calculate the BET specific surface area using adsorption data. The electrolyte contact angles were captured by an optical contact‐angle measuring device (Dataphysics OCA20).

##### Theoretical Simulation

All of the spin‐polarized DFT calculations within the generalized gradient approximation (GGA) in the Perdew–Burke–Ernzerhof (PBE) formulation were carried out using the Vienna Ab initio Simulation Package (VASP). A converged cutoff of 400 eV was used. The residual force on each atom was smaller than 0.05 eV Å^−1^ for structural relaxations. For the k‐point integration within the Brillouin zone, Monkhorst–Pack grid (2 × 2 × 1) was selected. A vacuum layer of 15 Å was used between continuous slabs.

## Conflict of Interest

The authors declare no conflict of interest.

## Supporting information

Supplementary Material

## Data Availability

The data that support the findings of this study are available from the corresponding author upon reasonable request.
