# Supplementary material for: CoFe2O4@rGO as a Separator Coating for Advanced Lithium–Sulfur Batteries
Source: Small Sci. 2023 Jun 27;3(8):2300045. doi: 10.1002/smsc.202300045 (PMC11936030; doi:10.1002/smsc.202300045)
Supplement: Supplementary file 1 — Supplementary Material [file SMSC-3-2300045-s001.pdf]

# Supporting Information

**$CoFe_2O_4$@rGO as a Separator Coating for Advanced Lithium–Sulfur Batteries**

*Yan Li, Jiabing Liu, Xingbo Wang, Xiaomin Zhang, Ning Chen, Lanting Qian, Yongguang Zhang*, Xin Wang*, and Zhongwei Chen**

Y. Li, X. Wang, X. Zhang, Prof. X. Wang
South China Academy of Advanced Optoelectronics & International International Academy of Optoelectronics at Zhaoqing, South China Normal University, Guangzhou 510006, China
E-mail: wangxin@scnu.edu.cn
J. Liu
State Key Laboratory of Reliability and Intelligence of Electrical Equipment, School of Materials Science and Engineering, Hebei University of Technology, Tianjin 300130, China
N. Chen
Canadian Light Source, Saskatoon, S7N 2V3, Canada
L. Qian
Department of Chemical Engineering, University of Waterloo, 200 University Ave. W, Waterloo, Ontario, Canada N2L 3G1
Prof. Y. Zhang
School of Materials Science and Engineering, State Key Laboratory of Reliability and Intelligence of Electrical Equipment, Hebei University of Technology, Tianjin 300130, China
E-mail: yongguangzhang@hebut.edu.cn
Prof. Z. Chen
Department of Chemical Engineering, University of Waterloo, Waterloo, ON N2L 3G1, Canada
E-mail: zhwchen@uwaterloo.ca

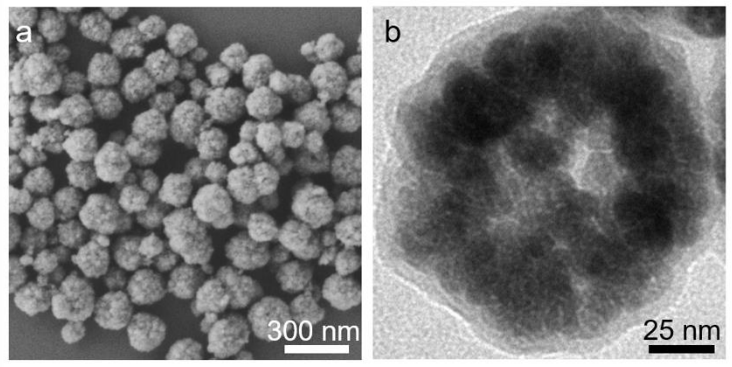


**Figure S1.** a) SEM image and b) TEM image of CFO.

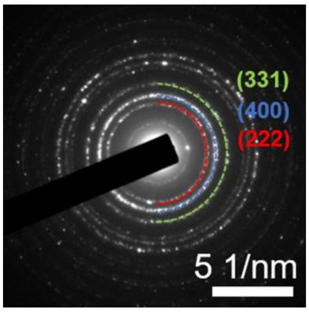


**Figure S2.** SAED pattern images of the CFO@rGO.

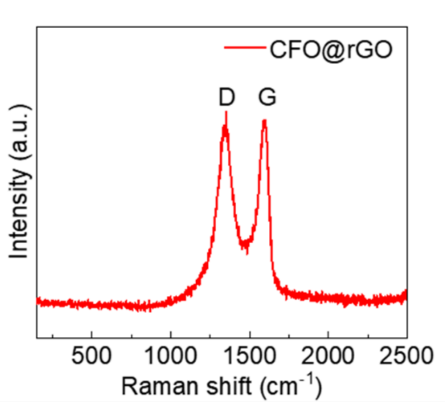


**Figure S3.** Raman spectrums of the CFO@rGO.

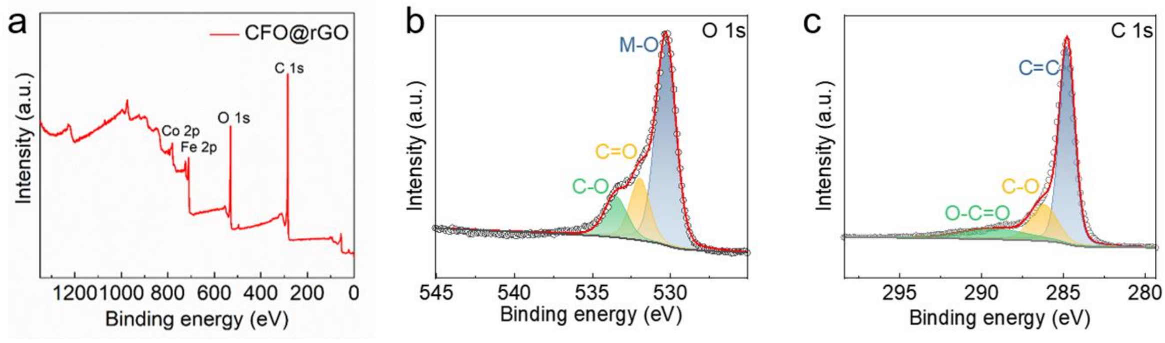


**Figure S4.** a) XPS survey scan of the CFO@rGO with the corresponding high-resolution scan of b) Co 2p and c) C 1s.

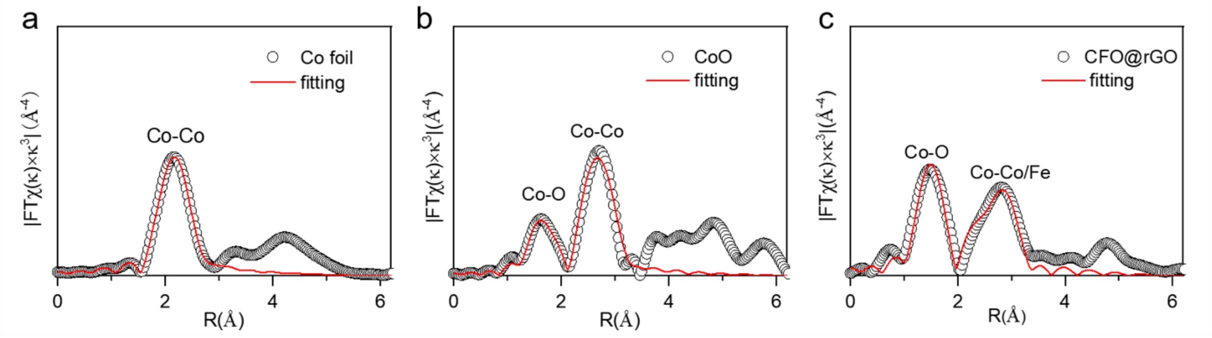


**Figure S5.** Fitting of fourier transform at Co K-edge of a) Co foil, b) CoO and c) CFO@rGO.

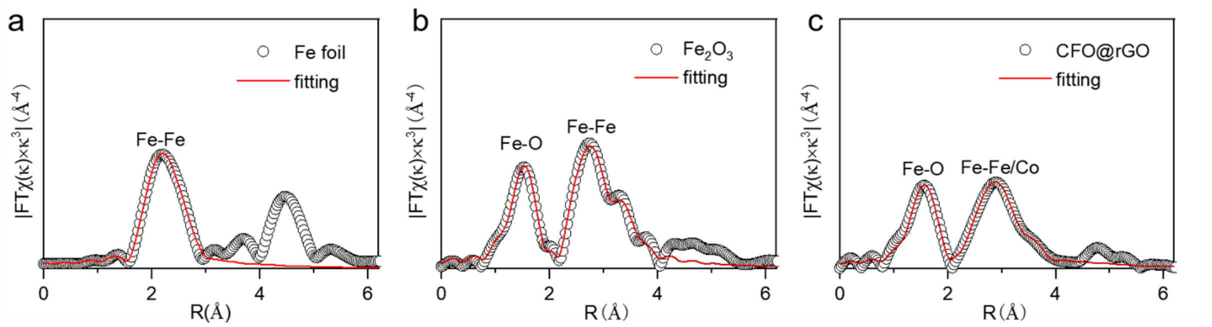


**Figure S6.** Fitting of fourier transform at Fe K-edge of a) Fe foil, b) $Fe_2O_3$ and c) CFO@rGO.

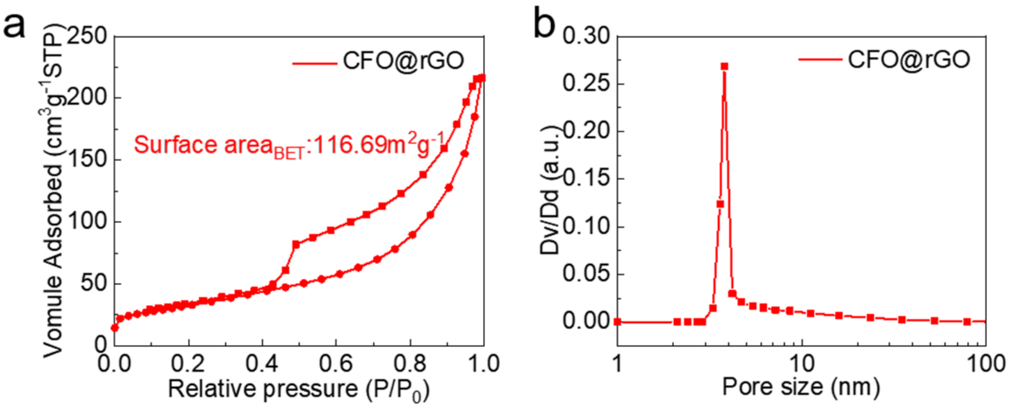


**Figure S7.** a) $N_2$ adsorption-desorption isotherms and b) the corresponding pore size distribution curves of the CFO@rGO.

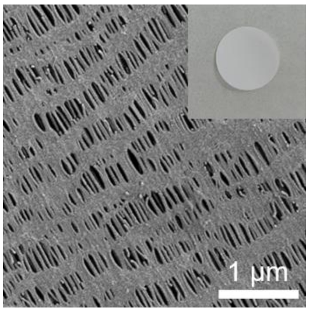


**Figure S8.** SEM image and photograph (inset) of PP separator.

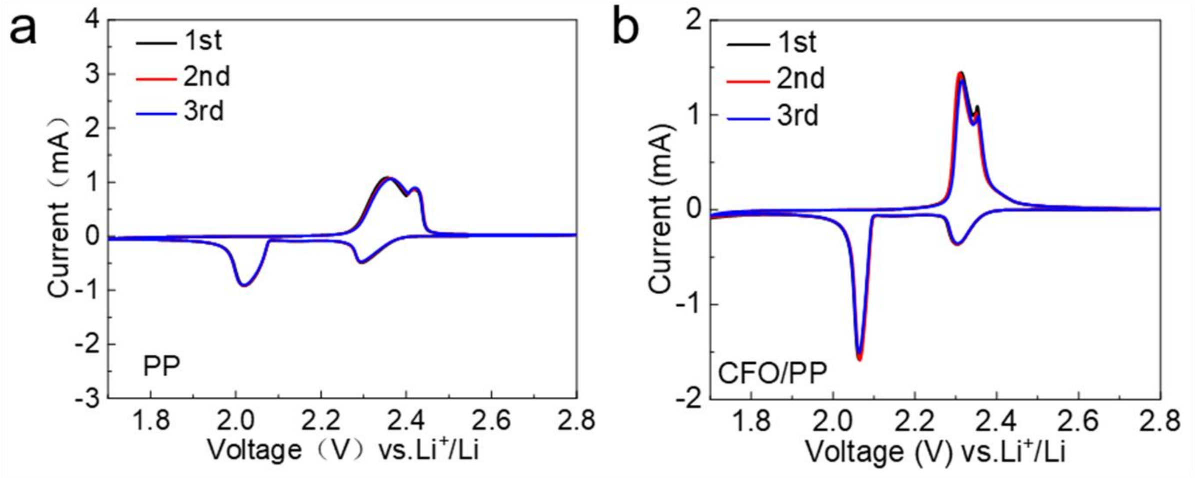


**Figure S9.** CV curves for cells with a) PP separator and b) CFO/PP separator.

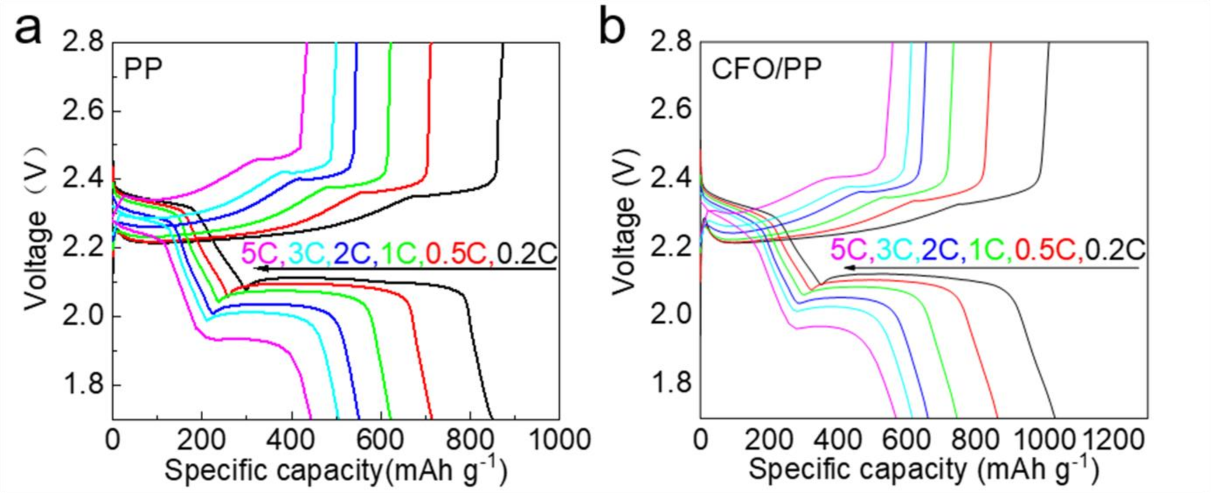


**Figure S10** Multi-rate voltage curves for a) PP separator and b) CFO/PP separator.

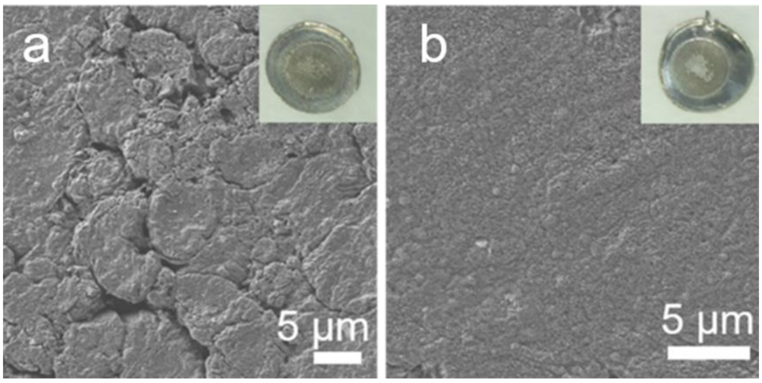


**Figure S11.** SEM images and photograph (inset) of a) PP separator and b) CFO@rGO/PP separator.

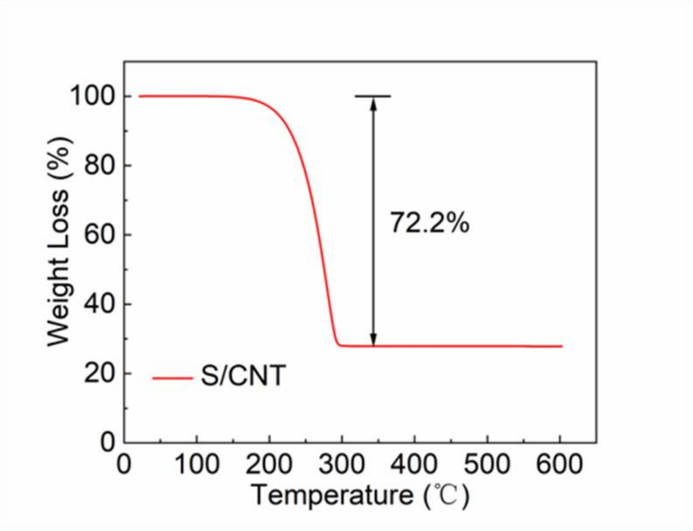


**Figure S12.** TGA curve of the S/CNT composite.

**Table S1.** EXAFS fitting parameters at the Co K–edge for various samples

| Sample | Shell | $CN^a$ | $R$(Å)$^b$ | $\sigma^2$(Å$^2$)$^c$ | $\Delta E_0$(eV)$^d$ | $R$ factor |
|---|---|---|---|---|---|---|
| Co foil | Co-Co | 12* | 2.49±0.01 | 0.0062±0.0003 | 7.0±0.4 | 0.0011 |
| CoO | Co-O | 6.0±1.5 | 2.10±0.01 | 0.0087±0.0038 | 11.0±2.2 | 0.0142 |
| | Co-Co | 12.2±2.9 | 3.00±0.02 | 0.0093±0.0017 | 9.1±1.5 | |
| CFO | Co-O | 6.0±1.5 | 1.97±0.01 | 0.0040±0.0032 | 4.1±1.4 | 0.0125 |
| | Co-Co/Fe | 5.3±1.8 | 3.06±0.01 | 0.0113±0.0041 | 9.7±1.0 | |

**Table S2.** EXAFS fitting parameters at the Fe K–edge for various samples

| Sample | Shell | $CN^a$ | $R$(Å)$^b$ | $\sigma^2$(Å$^2$)$^c$ | $\Delta E_0$(eV)$^d$ | $R$ factor |
|---|---|---|---|---|---|---|
| Fe foil | Fe-Fe1 | 8* | 2.46±0.01 | 0.0037±0.0033 | 5.2±2.2 | 0.0025 |
| | Fe-Fe2 | 6* | 2.85±0.01 | 0.0068±0.0048 | 4.1±1.5 | |
| $Fe_2O_3$ | Fe-O | 6.0±0.7 | 1.97±0.01 | 0.0117±0.0018 | 9.1±0.6 | 0.0059 |
| | Fe-Fe1 | 6.7±1.1 | 2.98±0.01 | 0.0083±0.0015 | 14.7±0.5 | |
| | Fe-Fe2 | 1.6±0.7 | 3.65±0.01 | 0.0032±0.0027 | 3.0±0.8 | |
| CFO | Fe-O | 4.8±0.5 | 1.99±0.01 | 0.0112±0.0016 | 9.4±0.5 | 0.0052 |
| | Fe-Fe/Co1 | 5.0±1.2 | 3.07±0.01 | 0.0110±0.0018 | 13.5±0.5 | |
| | Fe-Fe/Co2 | 2.3±1.1 | 3.57±0.01 | 0.0108±0.0033 | 14.2±0.6 | |
